# Enhancing parenteral therapy for anaemia among pregnant women in rural India

**DOI:** 10.5588/pha.26.0041

**Published:** 2026-08-24

**Authors:** P. Kotni, G. Ramaswamy, S. Cheela, S. Patil, P. Chinnakali, J.P. Tripathy, M. Khandale, D. Kamban, C. Konguvel, M. Manohar

**Affiliations:** 1 Department of Community Medicine and Family Medicine, AIIMS Bibinagar, Bibinagar, India;; 2 Department of Preventive and Social Medicine, JIPMER Pondicherry, Pondicherry, India;; 3 Department of Community Medicine, All India Institute of Medical Sciences Nagpur, Nagpur, India;; 4 Department of Public Health and Community Medicine, Tufts University School of Medicine, Boston, MA, USA;; 5 District Medical and Health Office, Government of Telangana, Yadadri Bhuvangiri, India.

**Keywords:** SORT IT, operational research, ferric carboxymaltose, Ganzoni formula, parenteral iron therapy

## Abstract

**SETTING:**

Primary care settings in a rural district of Telangana, India, under an anaemia control programme.

**OBJECTIVE:**

To explore the facilitators and barriers to parenteral iron therapy for moderate and severe anaemia, and possible solutions from the perspective of health care providers and pregnant women.

**DESIGN:**

A qualitative descriptive study. Twenty in-depth interviews and four focus group discussions were conducted with health care providers (primary care and district hospital) and postnatal mothers who had moderate or severe anaemia. Manual content analysis with inductive coding was done to identify themes and sub-themes.

**RESULTS:**

Facilitators included availability of IV iron at higher-level facilities, support from ASHAs (community health volunteers), community nutrition schemes, and growing access to digital health information. Barriers clustered around medicine stock-outs at lower-level facilities, transport difficulties, out-of-pocket costs, wage loss, need for escorts to hospitals, and strong fears and misconceptions about injections. Suggested solutions focused on uninterrupted IV iron supply, decentralised service delivery, capacity-building of frontline workers, digital and community-led communication strategies, and social support mechanisms.

**CONCLUSION:**

Strengthening supply chains, decentralising parenteral iron therapy, and enhancing culturally sensitive counselling and community support are essential to improve uptake and completion of parenteral therapy in primary care settings.

## Graphical abstract

**Figure:**
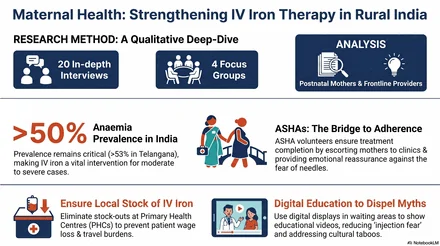

Anaemia affects nearly 37% of pregnancies globally.^1^ The deleterious effects of anaemia are inter-generational, with debilitating and long-lasting consequences for both expecting mothers and their children.^2,3^ According to the World Health Organization, anaemia is a major public health problem in several countries with prevalence >40%.^4^ The Global Nutrition Targets 2025 called for a 50% reduction in anaemia among women of reproductive age from 2012 baseline, but due to insufficient global progress, this target has been extended to 2030.^5^ Anaemia affects more than 50% of India’s population, particularly women and children. National Family Health Surveys (NFHSs) indicate that prevalence of anaemia among pregnant women in India was 50.3% and 52.2% in 2015–16 (NFHS-4) and 2019–21 (NFHS-5), respectively. In Telangana, a state in south-central India, the prevalence increased from 48.2% to 53.2% during the same period.^6,7^ As part of ongoing efforts to control anaemia, the Government of India launched Anaemia Mukt Bharat (AMB) programme in 2018.^8^ Notably, the AMB programme issued national guidelines for the use of intravenous iron sucrose (IVIS) or ferric carboxymaltose (FCM) for management of moderate and severe anaemia in pregnancy, in addition to the standard regimen of oral iron and folic acid (IFA) tablet for those with mild anaemia. These guidelines were implemented at all primary health centres (PHCs) in Telangana since 2018.

In public health settings in India, the implementation of IV iron regimens faces several practical and systemic challenges. FCM, although allows higher single-dose administration and fewer visits, is not widely available in the public sector, leaving IVIS as the predominant formulation used in most government facilities. On other hand, IVIS is administered with a maximum single-dose limit of 200 mg given no more than three times per week. The low-dose schedule requires multiple clinic visits for infusion, thus, limiting adherence to parenteral therapy.^9^ In addition, the requirement to calculate the total iron deficit using the Ganzoni formula before initiating IV iron therapy introduces another layer of complexity for clinicians, especially for primary care physicians working in resource-constrained high-volume settings.^10^

It is important to understand the complex, context-specific barriers and facilitators that are not easily captured by surveys.^11^ Previous studies on IV iron therapy implementation in low- and middle-income settings have emphasised the value of qualitative exploration in understanding acceptability, feasibility, and health-system challenges that could not be adequately explored using routine programme data.^12,13^ Hence, for this study, we adopted a qualitative design, to explore the facilitators and barriers to the use of parenteral iron therapy and possible solutions for improved uptake and completion of therapy from the perspective of health care providers (HCPs) and pregnant women in a rural district of Telangana, India.

## METHODS

A qualitative descriptive design involving in-depth interviews (IDIs) and focus group discussions (FGDs) with pregnant women and HCPs.

### Study setting

The study was conducted in Yadadri Bhuvanagiri district of Telangana, India. PHCs serve as first point of contact for maternal and child health services and play a crucial role in implementing anaemia control programmes like AMB. Each PHC provides essential health services including antenatal care (ANC), anaemia screening, and treatment in line with national guidelines. Auxiliary nurse midwives (ANMs) and ASHA workers (community health volunteers) provide community-level follow-up services.

### Management of anaemia in primary care setting

Pregnant women diagnosed with anaemia are managed as per AMB guidelines. Haemoglobin levels of 10–10.9, 7–9.9, and <7 gm/dl were classified as mild, moderate, and severe anaemia, respectively.^8^ At the village level, ANMs and ASHAs provide iron tablets, advise on locally available iron-rich foods, and monitor pregnant women with mild or moderate anaemia. Women with severe anaemia are referred to the PHC for management. Those who are not compliant with oral iron (mild/moderate anaemia and gestational age ≤34 weeks), IVIS (with a single maximum dose of 200 mg of iron not more than three times a week) or FCM is recommended at the time of diagnosis of mild or moderate anaemia. Those with mild or moderate anaemia and gestation age >34 weeks, IVIS is the recommended treatment. Severe anaemia (haemoglobin 5–6.9 gm/dl) and women <28 weeks of gestation are treated with IVIS or FCM at PHCs.^10^ These details are updated in the ‘Mother and Child Health’ portal.

### Study population and study period

HCPs (medical officers of PHCs, mid-level health providers/community health officers at sub-centres, ANMs, ASHA workers, and obstetricians from tertiary care centres) involved in the management of anaemia and postnatal mothers who had moderate or severe anaemia during pregnancy during the reference period (January–December 2024) at two selected PHCs were included. IDIs and FGDs were conducted during June–December 2025.

### Sampling

We used purposive sampling to select HCPs who were vocal and actively involved in the management of anaemia. A total of 12 IDIs were conducted with HCPs (medical officers – 5, community health officers – 3, and obstetricians – 4). Four FGDs were conducted; two each with ANMs and ASHA workers. Eight IDIs were conducted with mothers who were selected using purposive sampling based on their diagnosis of moderate or severe anaemia during the antenatal period and receipt of treatment advice at PHCs.

### Data collection

Investigators PK and SC, both fluent in local language (Telugu), conducted all IDIs and FGDs. SC had expertise in qualitative and mixed-methods research, while PK was trained in interview techniques. Both were female researchers with prior experience in anaemia in pregnancy and were not involved in programme implementation. Interviews were conducted at locations convenient to participants, including health facilities or homes. Separate pilot-tested interview guides with probes were used for HCPs and pregnant women to ensure consistency while allowing flexibility. Interviews were audio-recorded after consent, and findings were debriefed for participant validation. FGDs were held at PHCs with 6–8 participants each. Average duration was 30 min (10–45) for IDIs and 48 min (30–70) for FGDs. Data collection continued until saturation was reached. No monetary compensation was provided.

Reflexivity played a key role in interpreting the findings. The researchers remained aware of their own perspectives and potential biases while interpreting the findings ensuring that the analysis reflected participants’ experiences and viewpoints.

### Ethical statement

The study proposal was approved by the Institute Ethics Committee of AIIMS Bibinagar, India (AIIMS/BBN/IEC/Oct/2025/831-R). Informed written consent was obtained from study participants.

### Data analysis

Transcripts were prepared in English on the day of interview using audio recordings and field notes to ensure accuracy and completeness. Data were analysed manually using inductive content analysis in ATLAS.ti. PK, experienced in public health and qualitative research, conducted primary coding and identified codes related to facilitators and barriers and suggested solutions for managing moderate and severe anaemia in PHCs. GR independently reviewed the coding and thematic interpretations to enhance rigour and reduce bias. Discrepancies were resolved through discussion and consensus. Similar codes were grouped into sub-themes and themes, and findings were continuously checked against transcripts to ensure accurate representation of participants’ perspectives. The findings were reported according to the ‘consolidated criteria for reporting qualitative research’ (COREQ) guidelines.^14^

## RESULTS

We systematically mapped facilitators, barriers, and context-specific solutions across health-system, community, and individual levels, reflected in the number of themes, sub-themes, and codes identified in each domain.

Facilitators were described under four themes: community-based support and outreach, health-system infrastructure and processes, nutritional support systems, and anaemia awareness initiatives involving a total of 9 sub-themes and 18 codes which are described in Table 1 and visualised in Figure 1. Availability of iron formulations (IVIS) at PHCs and FCM at district hospitals, and access to blood transfusion at higher facilities were identified as important facilitators for timely management of moderate and severe anaemia.

**TABLE 1.** Facilitators of intravenous iron therapy for the management of moderate and severe anaemia in pregnant women in primary health centres of a district of Telangana, India, 2024–2025.

Theme/sub-theme/inductive codes (facilitators)	Verbatims
Community-based support and outreach	
Frontline worker engagement	
Regular home visits by ASHAs	ASHAs follow up by going to their house. She visits the home and asks how they are after taking the injection. She follows up and gives us an intimation through a call or by coming to the sub-centre. If there is any complication, we go and see, and after seeing, we directly refer to the PHC. – Medical officer (PHC)
Accompanying women for hospital visits	We have to go with them. Some are scared of injections. We tell them, ‘It’s just like a saline bottle, nothing will happen’. We sit with them until it is finished. – ASHA worker
Family and social involvement	
Involvement of husband and mother-in-law in counselling sessions to ensure home-level support	If their mother-in-law or mother comes with the patient, we tell them- what precautions she should take, how she should take, when to consult a doctor, food restrictions. – Medical officer (PHC)
Health-system infrastructure and processes	
Early detection and screening	
High rates of early registration in the first trimester (within 12 weeks)	Most of them in the first trimester. Like as soon as we are finding out that the woman is pregnant, we ask them to get registered. – ANM
Clinical treatment protocols	
Free distribution of IFA and calcium tablets.	We have iron folic acid (IFA) tablets and calcium tablets – I mean stock will be there, sir. No problem with the drug availability.
Availability of IV iron formulations at PHCs	Otherwise, we are having FCM facility also here, ferric carboxymaltose is also available. So, we are giving FCM injections to the antenatal mother also. – Specialist doctor (tertiary hospital)
	I used to go there for injections. I also received blood twice at the PHC. – Pregnant woman
Digital monitoring	
Regular data entry into the MCH/MCTS (programme) portal	Once they come to us, we take their Aadhaar cards and passbooks and enter the treatment details into the MCH portal. – Asha worker
Nutritional support systems	
Government nutrition schemes	
Provision of free milk and eggs through Anganwadi centres	Yes. Leafy vegetables, eggs... they give all those in the Anganwadi centre in the village. – Pregnant woman
Dietary counselling	
Specific guidance on iron-rich indigenous foods	She told me to eat well – leafy vegetables, fruits, and to take my medicine. – Pregnant woman
Anaemia awareness initiatives	
Community-based prevention	
Health awareness drives conducted during village-level health meetings	We are doing some health activities in gram sabhas and regular health days. We will tell them to take iron-rich diet and balanced diet, eat right concept, all of these. – CHO
Digital education outreach	
Use of social media, digital displays, and short educational videos in clinic waiting areas	Like social media or something, they are very much aware of these – knowledge levels are very good in pregnant women. They know by social media how to take the food, how to take the medicine. As the information is very much available, it is getting better now. – CHO

CHO = community health officer; MCH = maternal and child health; MCTS = Mother and Child Tracking System; PHC = primary health centre; IV = intra venous; ANM = auxiliary nurse midwife; ASHA = accredited social health activist; FCM = ferric carboxymaltose.

**FIGURE 1.**
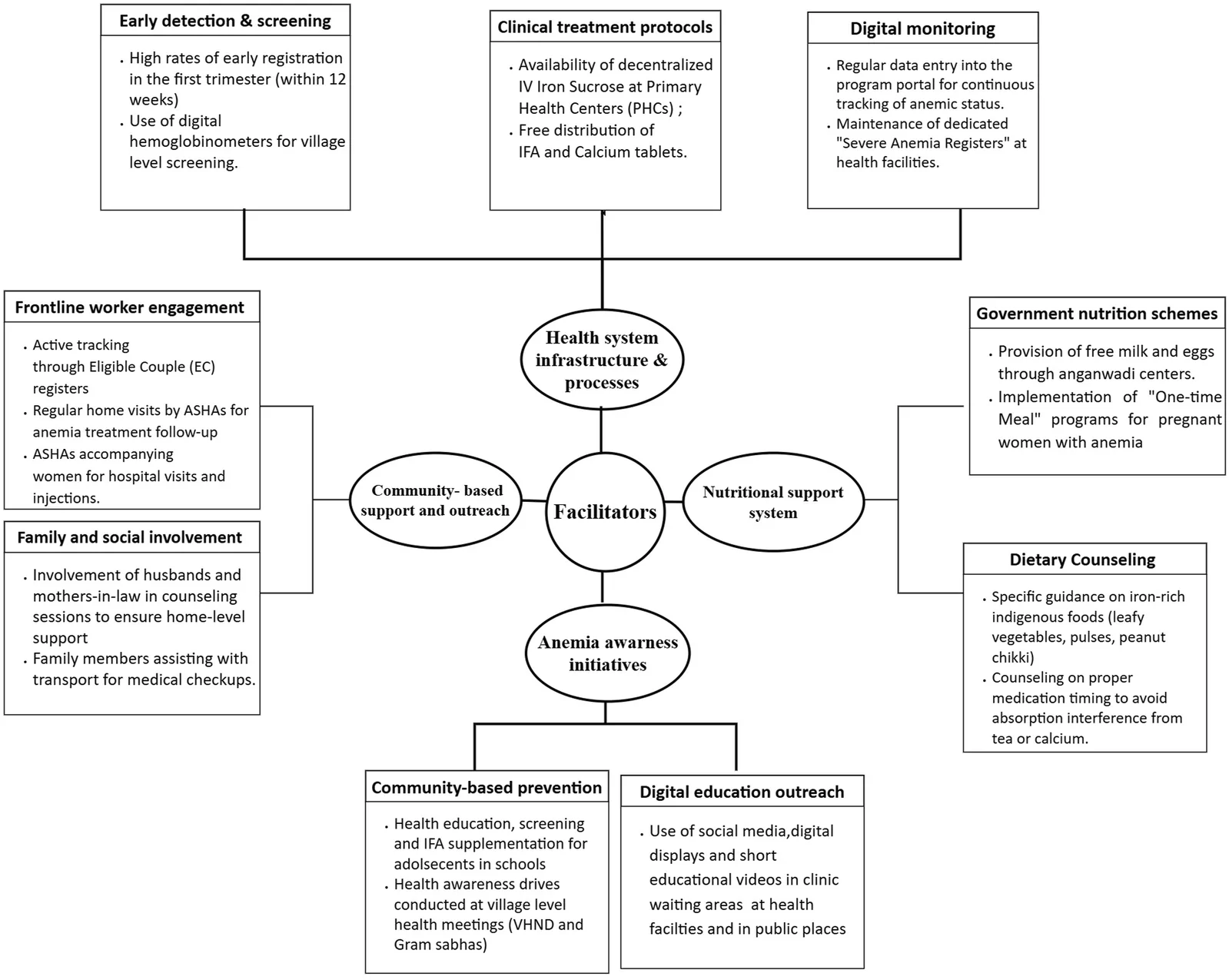
Facilitators of intravenous iron therapy for the management of moderate and severe anaemia in pregnant women in primary health centres of a district of Telangana, India, 2024–2025. IFA = iron and folic acid; ASHA = accredited social health activist.

ASHAs play a central role in supporting parenteral iron therapy by conducting regular home visits after each injection, monitoring for side effects, counselling women who are fearful of injections, and accompanying them to facilities, thereby improving completion of the prescribed IV iron schedule. An ASHA worker said:

We have to go with them. Some are scared of injections. We tell them, ‘It’s just like a saline bottle, nothing will happen’. We sit with them until it is finished.

Publicly funded community nutrition schemes via Anganwadi centres provide eggs, milk, and leafy vegetables, supporting dietary improvement alongside parenteral iron therapy. A pregnant woman highlighted the support provided through Anganwadi centres:

Yes. Leafy vegetables, eggs ... they give all those in the Anganwadi centre in the village.

Pregnant women also reported receiving dietary counselling on consuming iron-rich indigenous foods and adhering to treatment. Increasing access to social media, and digital educational content in clinics, is another facilitator which helps raise awareness and promote adherence to anaemia management. A community health officer (CHO) noted:

*…*knowledge levels are very good in pregnant women. They know by social media how to take the food, how to take the medicine.

Table 2 and Figure 2 describe health-system and patient-level barriers to completing parenteral therapy. Three themes emerged under barriers with 8 sub-themes and 21 codes. Frequent stock-outs of IV iron preparations at PHCs and complete non-availability at sub-centres force women to travel repeatedly to higher facilities for each dose. A community health officer (CHO) highlighted this challenge stating:

No injections are available, sir… We will send them to PHC only.

**TABLE 2.** Barriers to intravenous iron therapy for the management of moderate and severe anaemia in pregnant women in primary health centres of a district of Telangana, India, 2024–2025.

Theme/sub-theme/inductive codes (barriers)	Verbatims
Health-system constraints	
Logistical and resource gaps	No injections are available, sir. As of now, there are no injections available at sub-centre level. We will send them to PHC only. – CHO
Frequent stock-outs of IV iron formulations	
Service delivery hurdles	Blood bank is also a reason because every blood group is not available here. sometimes ... what I know. like AB blood groups are not available here. – Specialist doctor (tertiary hospital)
Lack of 24/7 blood storage at secondary levels	
Referral challenges	There won’t be transport, no buses. And again, having to travel while pregnant, my husband is not here, my family is not here, should I go for just one injection, what will happen if I stay. – Pregnant woman
Lack of reliable transport in remote hamlets	
High cost of private transport for referral visits	If the 102 vehicle (free maternal transport) is not available that day, they have to spend money for private vehicle. – ASHA worker
Patient-related barriers	
Treatment adherence and side effects	They say- sister, my stomach is hurting, or I am getting black motions. Some say they feel like vomiting. – ANM
Intolerance to oral iron therapy	
Awareness and behaviour	If severe anaemic also, mothers will not cooperate with us. If we suggest them to go to PHC and have iron sucrose injections regularly, some will go regularly, some will be like not willing to go and they neglect it. – CHO
Negligence toward follow-up visits	
Socio-cultural and economic factors	
Economic constraints	They have to come on time and get injections.. no one is available to accompany her. The husband goes to work, the mother-in-law looks after chores, she is alone. – ANM
Daily wage loss when attending clinics	
Household dynamics	It is mainly the daily wage workers or migrant workers who may only come for one or two visits and then lost to follow-up; that is an issue. – Medical officer (PHC)
Migration for work (brick kilns/construction) leading to loss of follow-up	
Myths and taboos	They said my blood was low and suggested Iron injections. I was very tense about whether they would suit me or not. My mother and in-laws also thought they might not suit me. – Pregnant woman
Belief that injections are ‘too strong’ for the body	
Fear of injections	Some people won’t prefer IV injections... That is stigma for some people, no injectable should be taken.. and they will avoid blood transfusions also some tribal people because that is from another source they will take orally, but they won't take blood. – Medical officer (PHC)

ANM = auxiliary nurse midwife; ASHA = accredited social health activist; CHO = community health officer; PHC = primary health centre.

**FIGURE 2.**
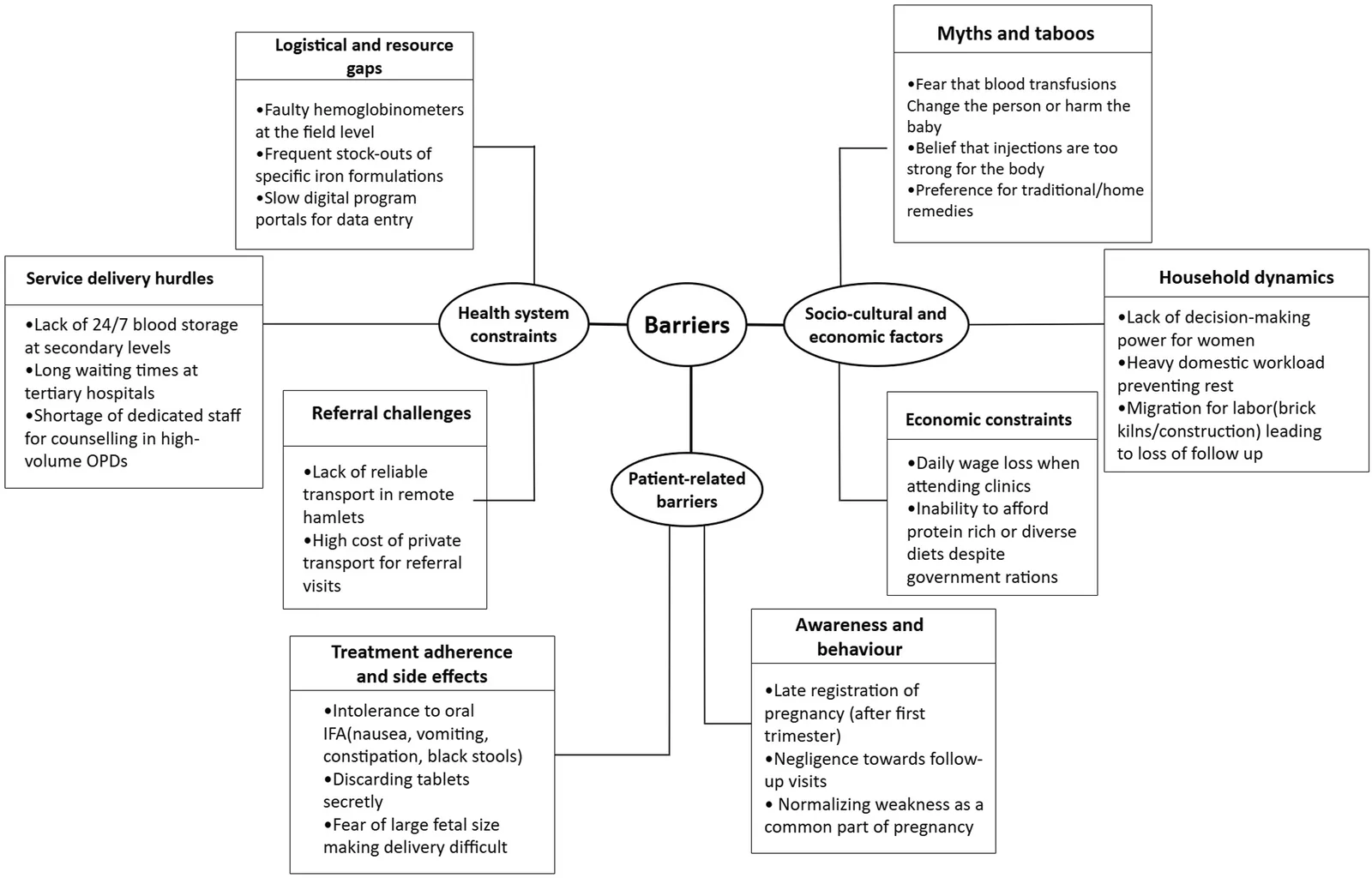
Barriers to intravenous iron therapy for the management of moderate and severe anaemia in pregnant women in primary health centres of a district of Telangana, India, 2024–2025.

For women in remote hamlets, lack of reliable public transport and non-availability of 102 service (free ambulance) on some days necessitate using private vehicles, adding considerable out-of-pocket expenditure. Multiple visits lead to wage loss for accompanying family members and create difficulties when no one is available to escort the pregnant woman. A pregnant woman shared:

There won’t be transport, no buses. And again, having to travel while pregnant, my husband is not here, my family is not here, should I go for just one injection, what will happen if I stay.

These economic and logistical constraints intersect with strong socio-cultural barriers. Many women and their families perceive injections as ‘too strong’, doubt whether they will ‘suit’ their body, and express a general fear of injections, especially in some tribal communities, leading to refusal or discontinuation of parenteral iron therapy.

Suggested solutions were summarised under four themes involving 8 sub-themes and 15 codes (Table 3). HCPs expressed that uninterrupted procurement and availability of IV iron and blood products across all levels, particularly 24/7 functional blood storage at secondary facilities and reliable IV iron stocks at PHCs, will improve management of anaemia. Decentralising services by administering iron injections at sub-centres under appropriate supervision is proposed to reduce travel barriers including costs. Capacity-building for ASHAs and ANMs on nutritional counselling, adherence support, and early warning sign recognition, along with streamlined referral processes to build trust and prioritise high-risk women, was suggested. Communication strategies suggested include using digital displays and videos in clinic waiting areas to strengthen anaemia literacy and treatment awareness to dispel misconceptions about iron injections. Providers felt that digital and visual media could make health communication more engaging and accessible for antenatal mothers. A specialist doctor from a tertiary hospital explained:

Nowadays everybody is interested or fond of digital media .... Counselling is one major part and displaying IEC material or videos during waiting time will be useful.

**TABLE 3.** Suggested solutions to intravenous iron therapy for the management of moderate and severe anaemia in pregnant women in primary health centres of a district of Telangana, India, 2024–2025.

Theme/sub-theme/inductive codes (solutions)	Verbatims
Health-system strengthening	
Infrastructure and equipment	
Ensuring 24/7 functional blood storage/banks at secondary level facilities	And below seven (haemoglobin), it is an indication for blood transfusion, so we refer them to the higher centre, either to the district hospital – CHO
Service decentralisation	
Administering iron injections at the sub-centre/village level under supervision to reduce patient travel	In the villages, all injections aren’t available, so we are bringing mothers to the PHC. If it’s in the villages, it’s even better. It would be very good if it’s done there. – ASHA worker
Capacity and process improvement	
Human resource development	
Specialised training for ASHAs/ANMs on nutritional counselling and identifying early warning signs	If there are some trainings for ASHAs, they will tell at the ground level only as of their knowledge. So, trainings will be very much useful. – CHO
Streamlined referrals	
Implementing a ‘slot-based’ referral system where patients are assured, they will see the same doctor at the higher facility to build trust.	Case segregation and the referral systems actually should be in proper way I think. That so that the load is reduced, only the high-risk patients will be given more importance, prioritised. – Specialist doctor (tertiary hospital)
Innovative communication	
Digital and visual literacy	
Using digital displays in clinic waiting areas to show nutritional videos.	Nowadays everybody is interested or fond of digital media. So if we can do as a small-small group, that is more much beneficial for the antenatal mothers. Counselling is one major part and displaying IEC material or videos during waiting time will be useful… – Specialist doctor (tertiary hospital)
Community-led monitoring	
Involving local leaders/village heads to mobilise transport for emergency or high-risk cases	But if it is a high-risk case, we don’t leave them. We talk to the sarpanch (village head) or arrange something. We make sure they go to hospital. – ASHA worker
Conducting ‘Godh Bharai’ (baby shower) ceremonies at centres to incentivise attendance.	These programmes like ‘Godh Bharai’ will be definitely useful for better management. – CHO

ANM = auxiliary nurse midwife; ASHA = accredited social health activist; CHO = community health officer; PHC = primary health centre.

HCPs highlighted the need for streamlined and better-coordinated referral systems to prioritise high-risk pregnancies, reduce overcrowding at higher facilities, and build patient trust through continuity of care. As one specialist doctor from a tertiary hospital said:

Case segregation and the referral systems actually should be in proper way I think. so that the load is reduced, only the high-risk patients will be given more importance, prioritised.

Few HCPs suggested community-led solutions involving village heads to mobilise transport for high-risk cases and organising culturally rooted initiatives such as Godh Bharai (pregnancy celebration) ceremonies at facilities or villages to encourage ANC attendance and improve engagement with maternal health services. As one ASHA worker noted:

But if it is a high-risk case, we don't leave them. We talk to the sarpanch (village head) or arrange something. We make sure they go to hospital.

## DISCUSSION

Our findings highlight that successful implementation of parenteral iron therapy for management of anaemia in pregnancy in primary care setting depends on multiple factors including community support, availability of parenteral therapy at PHCs, and socio-cultural beliefs. Our data add depth from a primary-care perspective in a rural setting.

At the community level, we found that ASHAs and family members reinforce women’s ability to complete schedule of parenteral iron therapy, by accompanying them to health facilities, and providing emotional reassurance when fear of injections is high and encouraging completion of all prescribed doses. In Malawi, similar enabling roles were played by spouses and mothers-in-law, who provided transport, food, and caregiving support.^15^ This support reduced the physical strain in later stages of pregnancy and helped women undertake long journeys to health facilities where parenteral iron therapy was provided. In Nigeria, husbands and matriarchs’ (‘domestic decision makers’) endorsement of parenteral therapy and financial support strongly shaped women’s willingness to accept parenteral iron therapy over oral IFA tablets.^16^ These studies suggest that community-based volunteers, ASHAs, and family networks are central to completion of all recommended doses of parenteral iron therapy. This implies that routine anaemia control programmes should strengthen the role of ASHAs/community volunteers through structured training materials, counselling tools, and incentives for travel and follow-up. Anaemia control programmes should take efforts to involve husbands and family members in counselling sessions.

In addition, increasing exposure to social media messages, digital displays, and educational videos in clinic waiting areas was perceived to improve awareness regarding dietary and non-dietary interventions for anaemia. Evidence from Nepal and other settings suggests that digital and social media–based counselling interventions can improve knowledge regarding iron-rich diets and healthy antenatal practices among pregnant women.^17,18^ These findings indicate that combining medical treatment with culturally appropriate health communication through digital or social media may improve treatment acceptance and adherence.

Health-system availability and accessibility of parenteral iron emerged as a major theme. In our context, when stocks were available (IVIS at PHCs and FCM at district hospitals), it facilitated management of moderate and severe anaemia. This is consistent with observational and qualitative work from India, where availability of iron sucrose, designated space for IV infusion, and trained staff were described as key facilitators of implementation, while stock-outs and complex referral pathways led to missed or incomplete therapy.^19^ Similar facilitators and barriers were reported in Australia during implementation of parenteral iron therapy.^13^

Our study findings highlight financial and logistical burden on women when IV iron formulations were not available at their nearest health facility. An ASHA worker highlighted this challenge, stating, ‘If the 102 vehicle (free maternal transport) is not available that day, they have to spend money for private vehicle’. This pattern is similar to findings from Malawi, where long distances, lack of transport, and long waiting times were major barriers for parenteral iron therapy.^15^ Similarly, a systematic review which used CFIR (consolidated framework for implementation research) identified ‘available resources’ as core constructs limiting implementation, and identified stock reliability, infrastructure, and staffing as priority targets for implementation strategies.^20^ Across settings, these converging findings suggest that scaling up parenteral iron therapy during pregnancy cannot rely solely on district-level services. Strengthening availability at the primary care level through consistent supplies of IV iron and related consumables within essential medicine supply chains, along with support for transport and outreach services, is critical. Where single-dose FCM is available, our findings, consistent with other studies, indicate that its shorter infusion time and single-visit regimen can reduce the overall burden on both pregnant women and care providers.

Community norms and cultural ideas about injections influence how people feel about starting treatment and whether they continue it. In our study, many women and families perceived IV iron injections as ‘too strong’, worried they might not ‘suit’ their body, and contributed to refusal or discontinuation of parenteral iron therapy. Fear of injections and taboo around blood transfusions in some native communities also contributed to hesitancy and poor acceptance of recommended therapies. The study from Malawi reported that participants similarly described myths linking IV iron with ‘satanism’, and harm to the unborn child.^15^ There were concerns expressed by the participants related to the colour of the treatment as a woman from Malawi in an FGD said: ‘Upon looking at the colour of the medication as it was red, I was worried. I thought that the medication was harmful, and can harm my child’.^21^ In Nigeria, stakeholders anticipated that rumours and misconceptions about parenteral iron therapy could arise, but high trust in HCPs and counselling during antenatal health talks could reduce such fears and misconceptions.^16^ In the study from Australia, HCPs reported anxiety about anaphylaxis and past bad clinical experiences with older versions of IV iron formulations and this prevented them prescribing even when newer products and guidelines were available.^13^ These findings highlight the need for culturally sensitive counselling and community engagement to address misconceptions and improve acceptance of parenteral iron therapy.

Our study has the following strengths. First, we captured the perspectives of different types of providers at both primary and tertiary care levels, as well as those of pregnant women receiving or eligible for parenteral iron therapy, providing a comprehensive view of implementation challenges and opportunities across stakeholder groups. Second, researchers were trained in qualitative research, which strengthened the study design, data collection, and analysis by ensuring the use of appropriate interviewing techniques, reflexive thematic analysis, and systematic coding procedures, and by enhancing the credibility and trustworthiness of the findings. We also followed COREQ guidelines for reporting the conduct and findings of the study.

Our study also had some limitations. First, it is context-specific, drawing on experiences from a selected district and health facilities, which may limit generalisability to other districts or states. Second, the study focused on users and immediate community stakeholders and does not include perspectives of higher-level policymakers, supply-chain managers, which may under-represent structural and policy barriers such as procurement decisions and financing constraints.

## CONCLUSION

Our findings are consistent with the published studies on implementation, reinforcing that parenteral iron in pregnancy is both feasible and acceptable when community support systems are mobilised, IV iron formulations and staff are reliably available, and socio-cultural concerns about injections are addressed using a family and community approach. Our data also document the overall economic and logistical burden of multi-dose IVIS in rural settings. These insights support implementation strategies that simultaneously strengthen supply chains, role of ASHAs and family caregivers, and awareness generation initiatives in the community.
